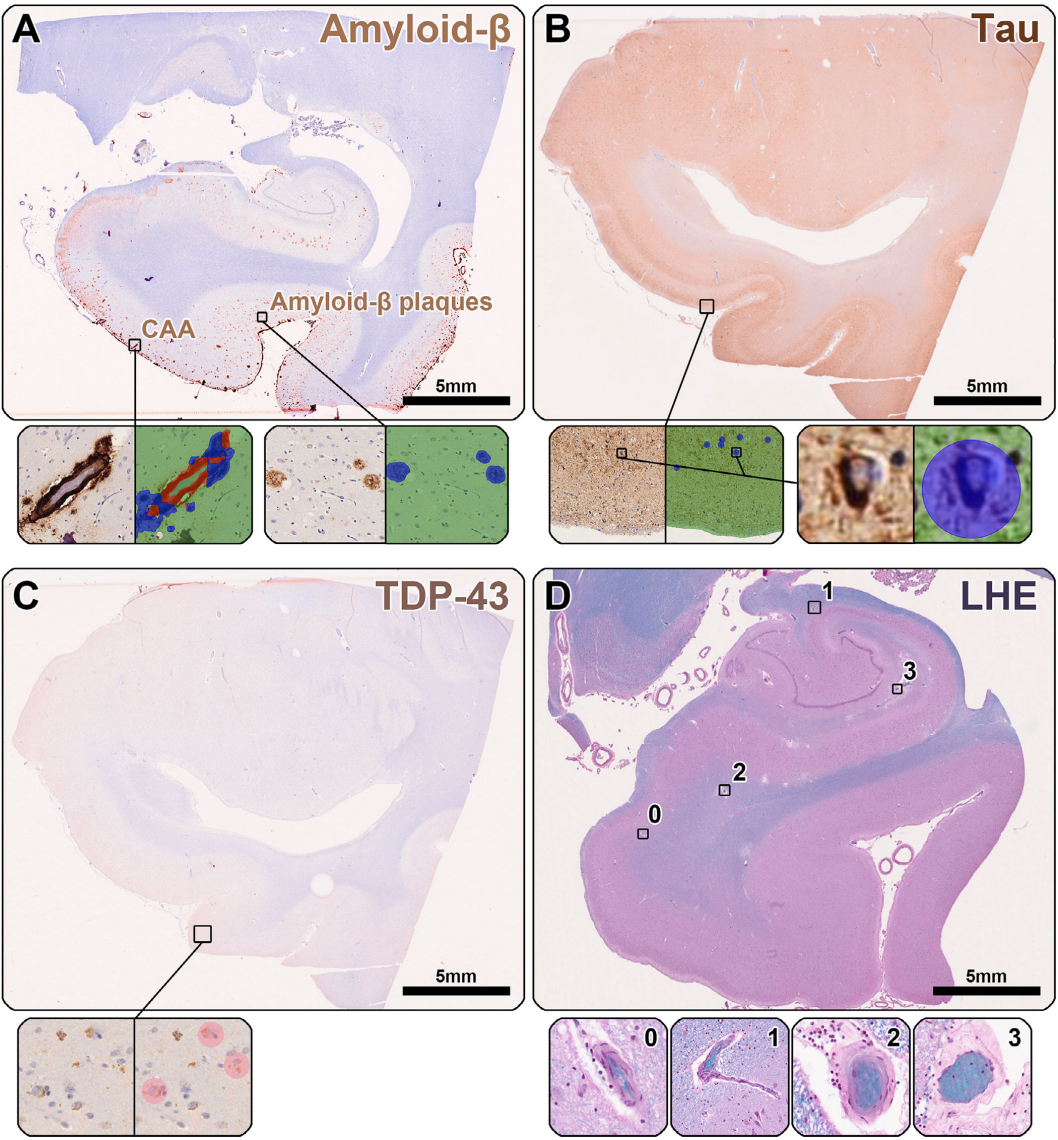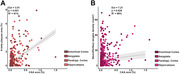# Links Between Microvascular and AD‐related pathology in the Medial Temporal Lobe

**DOI:** 10.1002/alz.085804

**Published:** 2025-01-03

**Authors:** Valentina Perosa

**Affiliations:** ^1^ Massachusetts General Hospital, Harvard Medical School, Boston, MA USA

## Abstract

**Background:**

Cerebral small vessel disease (CSVD), which includes cerebral amyloid angiopathy (CAA) and arteriolosclerosis, often co‐occurs with Alzheimer’s disease (AD) pathology. The medial temporal lobe (MTL) is susceptible to hosting multiple AD pathologies, such as neurofibrillary tangles (NFTs), amyloid‐β plaques, phospho‐Tar‐DNA‐Binding‐Protein‐43 (pTDP‐43), as well as CSVD. Whether a causal relationship between these pathologies exists remains largely unknown, but one potential linking mechanism is the dysfunction of perivascular clearance. Our objective was to examine the burden of CSVD in the MTL of a pathological AD cohort and to establish the associations between CSVD and AD‐related pathologies, as well as between CSVD and enlarged perivascular spaces (EPVS), a potential indicator of clearance dysfunction.

**Method:**

The study included 156 autopsy cases (mean age at death 79.4±10.9 years, 90 females) from the Massachusetts Alzheimer’s Disease Research Center (MADRC). One hemisphere was preserved in formalin, and 5 µm‐thick sections were cut from predefined regions of the hippocampal body and entorhinal cortex. These sections were subsequently stained using luxol fast blue with hematoxylin&eosin (LHE), and antibodies against amyloid‐β, hyperphosphorylated tau (At8), and pTDP‐43, following standard histological and immunohistochemical protocols. Utilizing deep‐learning models (Aiforia®), we computed the burden of CAA, amyloid‐β plaques, NFTs, and pTDP‐43 inclusions (Figure 1). Additionally, the severity of arteriolosclerosis and the % area of EPVS were evaluated on the LHE sections.

**Result:**

In linear mixed effects models CAA was positively associated with the density of NFTs (Est = 7.21; p = 0.024; R^2^ = 69%) and amyloid‐β plaque burden (Est = 3.01; p<0.001; R^2^ = 61%) in all regions of interest. Arteriolosclerosis had no direct effect on parenchymal AD‐related pathologies but showed a positive interaction with CAA in the association with PVS enlargement. There was no relationship between pTDP‐43 inclusions and arteriolosclerosis.

**Conclusion:**

These results point towards an association between microvascular pathology and AD‐related pathology, possibly mediated by clearance dysfunction.